# Impacts of Wastewater Treatment Processes on the Occurrence Characteristics of Microplastics—Taking Four Wastewater Treatment Plants in Northern Henan Province as Examples

**DOI:** 10.3390/toxics14090775

**Published:** 2026-08-31

**Authors:** Yiping Guo, Jinhong Li, Shihang Ni, Qianqian Zhang, Li Guo, Bingtao Liu, Peng Liu, Weigao Zhao, Yupeng Li, Hanzhong Jia

**Affiliations:** 1 School of Ecology and Environment, North China University of Water Resources and Electric Power, Zhengzhou 450046, China; 2 Henan Engineering Research Center of Water Pollution and Soil Damage Remediation, Zhengzhou 450046, China; 3 Environmental Monitoring and Safety Center, Department of Ecology and Environment of Henan Province, Zhengzhou 450046, China; 4 College of Natural Resources and Environment, Northwest A&F University, Xianyang 712100, China; 5 Department of Environmental Engineering, School of Environmental Science and Engineering, Tianjin University, Tianjin 300072, China; 6 Henan Provincial Urban and Rural Water Affairs Research Institute Co., Ltd., Zhengzhou 450046, China

**Keywords:** wastewater treatment plants, process configuration, microplastics, occurrence characteristics, morphological changes, removal efficiency

## Abstract

As emerging persistent contaminants, microplastics (MPs) are ubiquitous in aquatic environments. Wastewater treatment plants (WWTPs) act as critical sinks and sources of MPs, and their MPs removal efficiency can strongly influence aquatic ecological safety. To investigate how different process configurations affect MPs occurrence and removal performance, four municipal WWTPs equipped with diverse biological and advanced treatment processes were sampled and analyzed. MPs were extracted via density flotation combined with H_2_O_2_ digestion, and their morphological and polymeric features were identified using stereomicroscopy and Fourier-transform infrared (FTIR) spectroscopy. The results revealed that MPs removal efficiencies differed significantly across treatment processes: WWTP4 exhibited the highest comprehensive removal efficiency of 63.89% in the operational situations, followed by WWTP1 of 56.25% and WWTP3 of 51.61%, while WWTP2 exhibited the weakest elimination capacity of 28.12%. It could also be concluded that the differences were primarily governed by biological adsorption and advanced filtration units. Meanwhile, Fragmented and pellet MPs showed higher removal efficiencies than fibrous and film-shaped MPs, while Polymers including PVC, PS, PTFE and PP were more resistant to removal. Longer hydraulic retention time (HRT) and a stable and sufficiently long sludge retention time (SRT) promoted the association of MPs with activated sludge, whereas excessive aeration induced MPs fragmentation and resuspension. Optimizing operational parameters and upgrading advanced filtration facilities could facilitate the regulation and management of MPs. This study provides a theoretical basis for mitigating MPs emissions from municipal WWTPs.

## 1. Introduction

Microplastics (MPs) generally refer to plastic particles smaller than 5 mm. As a category of emerging persistent contaminants, they are widely distributed in diverse aquatic media, such as oceans, rivers, lakes and groundwater [1,2,3]. Owing to their tiny particle size, high chemical stability, strong migration and bioaccumulation potential, MPs not only disrupt the structure and function of aquatic ecosystems via organism ingestion, pollutant adsorption and physical blockage, but also pose latent hazards to human health through food chain transmission [4,5,6]. Therefore, MP pollution has become an important research focus in environmental science and engineering.

Environmental MPs derive from two major categories: primary and secondary MPs [7]. Primary MPs mainly originate from particles released directly or leaked from personal care products and industrial plastic pellets. Secondary MPs are gradually generated from large plastic products under physical abrasion, ultraviolet radiation, chemical oxidation and biodegradation [8]. These MPs enter WWTPs through surface runoff, industrial wastewater discharge and domestic sewage, making WWTPs important nodes for both MP retention and release for the migration and transformation of environmental MPs [9]. On one hand, WWTPs intercept and remove MPs through physical interception, biological adsorption and sedimentation. On the other hand, MPs retained in final effluent and excess sludge may still be discharged into natural water bodies or agricultural soil during sludge disposal, thereby causing secondary environmental contamination [10].

Previous studies in China and elsewhere on MPs in WWTPs mainly focus on MPs occurrence profiles and removal efficiency in a single urban sewage treatment system. For instance, previous investigations reported that the influent MPs concentration of a municipal WWTP in Beijing reached 15.46 n·L^−1^, which decreased to 0.3 n·L^−1^ after full treatment, corresponding to a total removal rate of 98.1% [11]. The influent MPs abundance of large-scale municipal WWTPs in Shanghai ranged from 171.89 to 226.27 n·L^−1^, with an average overall removal efficiency of approximately 60% [12]. Existing literature indicated that MPs in urban WWTPs were predominantly fibrous and fragmented, accounting for over 60% of total MPs, and their particle sizes were mostly concentrated within 20–500 μm; meanwhile, transparent and colored MPs dominated the color composition, while PE, PP and PET were the dominant polymer types [3,9]. However, data revealed that the MPs removal efficiency of some township WWTPs ranged only from 18.8% to 54.4% [13]. In terms of treatment performance, primary facilities (e.g., bar screens and grit chambers) can eliminate 40–60% of MPs, and secondary biological units (e.g., A^2^/O and SBR processes) further remove 20–40% of residual MPs, while tertiary advanced treatment (e.g., filtration and constructed wetlands) contributes limited additional removal [10]. Besides, surveys have demonstrated that industrial wastewater contained MPs at concentrations up to 353.26 n·L^−1^, dominated by fibrous shapes; by contrast, bead-like MPs accounted for 60.24% of total MPs in domestic sewage [14]. Meanwhile, sludge acts as a major MPs sink, with MPs concentrations reaching 4.4 × 10^3^–9.2 × 10^3^ n/kg (dry sludge) [15]. Although prior research has laid a foundation for understanding MPs distribution and removal mechanisms in urban WWTPs, further investigations of MPs pollution profiles under varied process flows in typical regional WWTPs are still lacking.

In this study, four urban WWTPs from northern Henan region with similia wastewater collection source and differentiated water treatment process routes were selected as study sites, by examining the differences in the abundances, shapes, and colors of MPs at various key stages across different processing techniques, hoped to systematically explore MPs occurrence characteristics and removal mechanisms in WWTPs with different treatment processes, and clarify the patterns and mechanisms of MPs removal in wastewater treatment systems. The findings could provide scientific support for optimizing operational parameters, elevating MPs removal efficiency and mitigating environmental MPs emissions.

## 2. Materials and Methods

### 2.1. Study Sites

**Figure 1:**
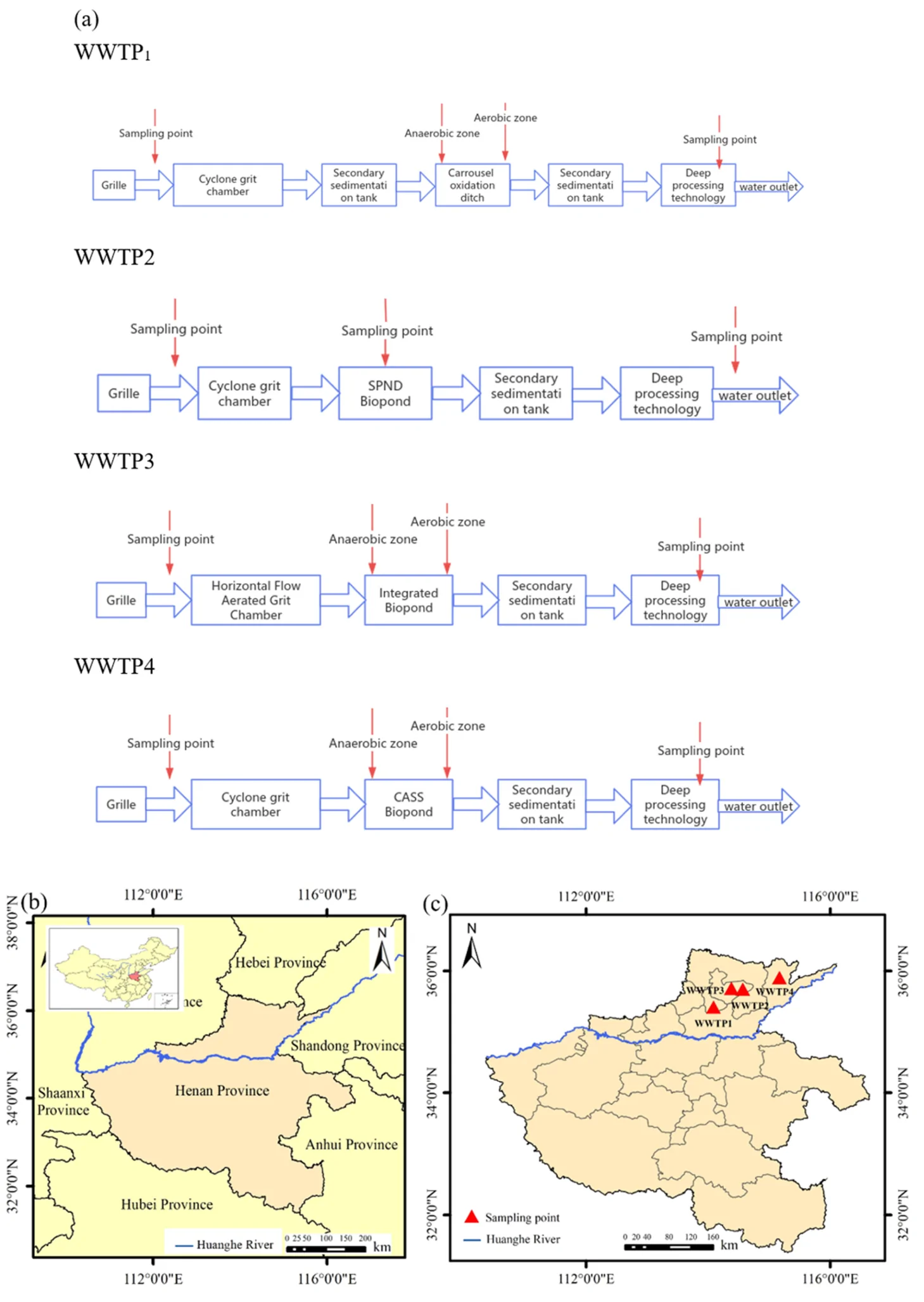
Process flow diagrams of four wastewater treatment plants (WWTP1 to WWTP4). (**a**) Process flow diagrams and sampling sites of four WWTPs; (**b**) Geographical location; (**c**) Detailed locations of the four WWTPs. Provincial boundary data were derived from Henan elevation map datasets. Sampling coordinates of the four WWTPs (red triangles) were obtained from the Amap Open Platform (https://lbs.amap.com/) merely for non-commercial academic research. National borders remain unchanged.

Four typical municipal wastewater treatment plants (WWTP1–WWTP4) in the northern Henan region were selected as study sites, all of which mainly treat municipal domestic sewage (Figure 1). All four WWTPs include core units such as pretreatment, biological treatment, advanced treatment, and disinfection, but there are differences in biological treatment configurations and advanced treatment methods, which provided a good comparative basis for comparing the migration, transformation, and removal effects of microplastics under different process conditions.

In general, WWTP1 adopts the carousel oxidation ditch process, while WWTP4 applies the sequencing batch CASS process, and WWTP2 and WWTP3 both operate continuous-flow biological pond treatment systems, where SPND Biochemical Reactor in WWTP2 includes Anaerobic Tank, Micro-aerobic Aeration Tank, and Aerobic Tank, while Integrated Biopond in WWTP3 means an integrated biological treatment tank, which is a multi-stage activated sludge nitrogen removal system, and each unit consists of an anoxic tank connected to an aerobic tank. The deep processing technology units of all plants cover multiple configurations, including deep filters, rotary disc filters and ozonation disinfection. Such distinct process layouts provide a basis for analyzing how different treatment schemes affect MPs removal efficiency and variations in particle size composition. The red hair cut in Figure 1a represents the sampling sites for each WWTP.

The specific operating conditions of each plant are as follows.

WWTP1 covers an area of 0.05 square kilometers, with a service area of approximately 20.58 square kilometers. Its designed total daily sewage treatment capacity is 50,000 tons, and primarily provides centralized wastewater treatment services for both municipal wastewater within the urban planning zone and wastewater from surrounding enterprises. The operation runs a 4-h cycle, that is 1 h of water inflow, 2 h of aeration and sedimentation, and 1 h of water discharge. The HRT is usually 30 s in the cyclone grit chamber process, while flow velocity is 0.02–0.1 m/s.

WWTP2 covers an area of 0.05 square kilometers, with a service area of approximately 17.7 square kilometers. Its designed total daily sewage treatment capacity is 20,000 tons, and primarily provides centralized wastewater treatment services for both municipal wastewater within the urban planning zone and wastewater from surrounding enterprises. The HRT is usually 8.4 h in SPND Biopond.

WWTP3 covers an area of 0.03 square kilometers, with a service area of approximately 9.78 square kilometers. Its designed total daily sewage treatment capacity is 25,000 tons, and primarily provides centralized wastewater treatment services for both municipal wastewater within the urban planning zone and wastewater from surrounding enterprises. The HRT is usually 3.96 h in the anaerobic zone, 13.4 h in the aerobic zone, and 4.5 h in the secondary sedimentation tank.

WWTP4 covers an area of 0.038 square kilometers, with a service area of approximately 13.9 square kilometers. Its designed total daily sewage treatment capacity is 15,000 tons, and primarily provides centralized wastewater treatment services for both municipal wastewater within the urban planning zone and wastewater from surrounding enterprises. The HRT is usually 1.5 h in the anaerobic zone, 11.6 h in the aerobic zone, and 4.5 h in the secondary sedimentation tank. There is a hydrolysis-acidification pretreatment process before the anaerobic zone, which could decrease the following anaerobic treatment time, and the HRT is usually 8.5 h.

### 2.2. Sample Processing and Detection

Samples were collected in October and November 2024, 4 times every month. The sampling method is identical to that used for conventional monitoring parameters at wastewater treatment plants, that is, a random sampling approach. The final data for each monitoring point is the average of eight measurements. All sampling was conducted on non-rainy days.

For detection, the density flotation method with saturated sodium chloride solution (ρ = 1.2 g/cm^3^) was used to extract microplastics from sewage. A 100 mL water sample was measured and placed in a 250 mL beaker, and 36 g of sodium chloride was added and stirred until completely dissolved. There were three 50-mL centrifuge tubes, and the supernatant was collected into an Erlenmeyer flask after centrifugation. Two of the centrifuge tubes and the original beaker were rinsed with 5 mL, 5 mL, and 10 mL of saturated sodium chloride solution in sequence, respectively. The rinsing solution was combined into the 1 remaining centrifuge tube, and the supernatant was collected after another round of centrifugation and merged into the same Erlenmeyer flask. Then, 20 mL of saturated sodium chloride solution was added to the centrifuge tube, centrifugation was repeated, and the supernatant was collected. Finally, all supernatants were combined.

The combined supernatant was vacuum-filtered through a 0.45 μm glass fiber filter membrane with a diameter of 50 mm. The suspended solids retained on the filter membrane were transferred to a 50 mL glass tube with 20 mL of 30% H_2_O_2_ solution, and the tube mouth was sealed with aluminum foil. After mixing uniformly, digestion was carried out at 60 °C for 24 h to completely remove organic matter. After digestion, the digested solution was vacuum-filtered again through a 0.45 μm glass fiber filter membrane, and the filter membrane was rinsed with 5 mL of absolute ethanol to accelerate water evaporation. The filter membrane was transferred to a glass Petri dish, dried to constant weight in an oven at 45 °C, removed and stored in the dark for subsequent analysis. The Table 1 provides detailed information on the reagents used in the experiment.

A stereomicroscope (Ouster Optical Instruments Co., Ltd.) was used to quantitatively count the microplastics in the sewage at a magnification of 100×. The abundance (n·L^−1^), shape, color, and particle size were recorded, and representative images were captured for typical microplastic types. A Fourier-transform infrared (FTIR) spectrometer (Thermo Fisher Scientific Inc.; iN10 MX) was used to identify and screen the polymer types of microplastics. MPs were determined in the spectral range of 675~4000 cm^−1^ using 16 scans in reflection mode. The polymer components were identified by OMNIC Picta software (Version 1.9) and matched with the established standard sample library. The polymer type was determined when the match score exceeded 60%. The Table 2 shows the details of the instruments used in the experiment.

The data of COD, NT, NH_3_-N, and NP were provided by WWTPs, and they were obtained according to national standard methods “Discharge Standard for Pollutants from Municipal Wastewater Treatment Plants” (GB 18918-2002) [16]. Therein, COD was tested through dichromate titration (GB 11914) [17], TN was tested through Alkaline potassium persulfate digestion–UV spectrophotometric method (HJ 636-2012) [18], NH_3_-N was tested through Distillation–Neutralization Titration Method (GB 7478) [19], and NP was tested through Ammonium molybdenum(IV) spectrophotometric method (GB 11893) [20].

### 2.3. Data Analysis Methods

Microsoft Excel 2020, Origin 2024 and ArcGIS10.8 were used for experimental data analysis, graph plotting and spatial analysis, respectively.

### 2.4. Quality Control

Strict quality control and quality assurance measures were implemented during the entire sampling and experimental process. Non-plastic containers were used whenever possible in all sampling and experimental procedures. All instruments and tools were thoroughly rinsed with ultrapure water before use and covered with aluminum foil when idle to prevent contamination. All personnel wore cotton lab coats, gloves, and masks throughout the operation.

To assess the level of laboratory background contamination, blank control experiments were conducted using ultrapure water as the matrix following exactly the same procedure as for real samples. The number of microplastics (MPs) detected in blank samples was subtracted from the counts of actual samples to correct for background contamination. During microplastic detection by micro-Fourier-transform infrared (FTIR) spectroscopy, the entire filter membrane was comprehensively examined to avoid analytical errors caused by the uneven distribution of microplastics on the membrane.

## 3. Results and Discussions

### 3.1. Identification of Polymer Components

**Figure 2:**
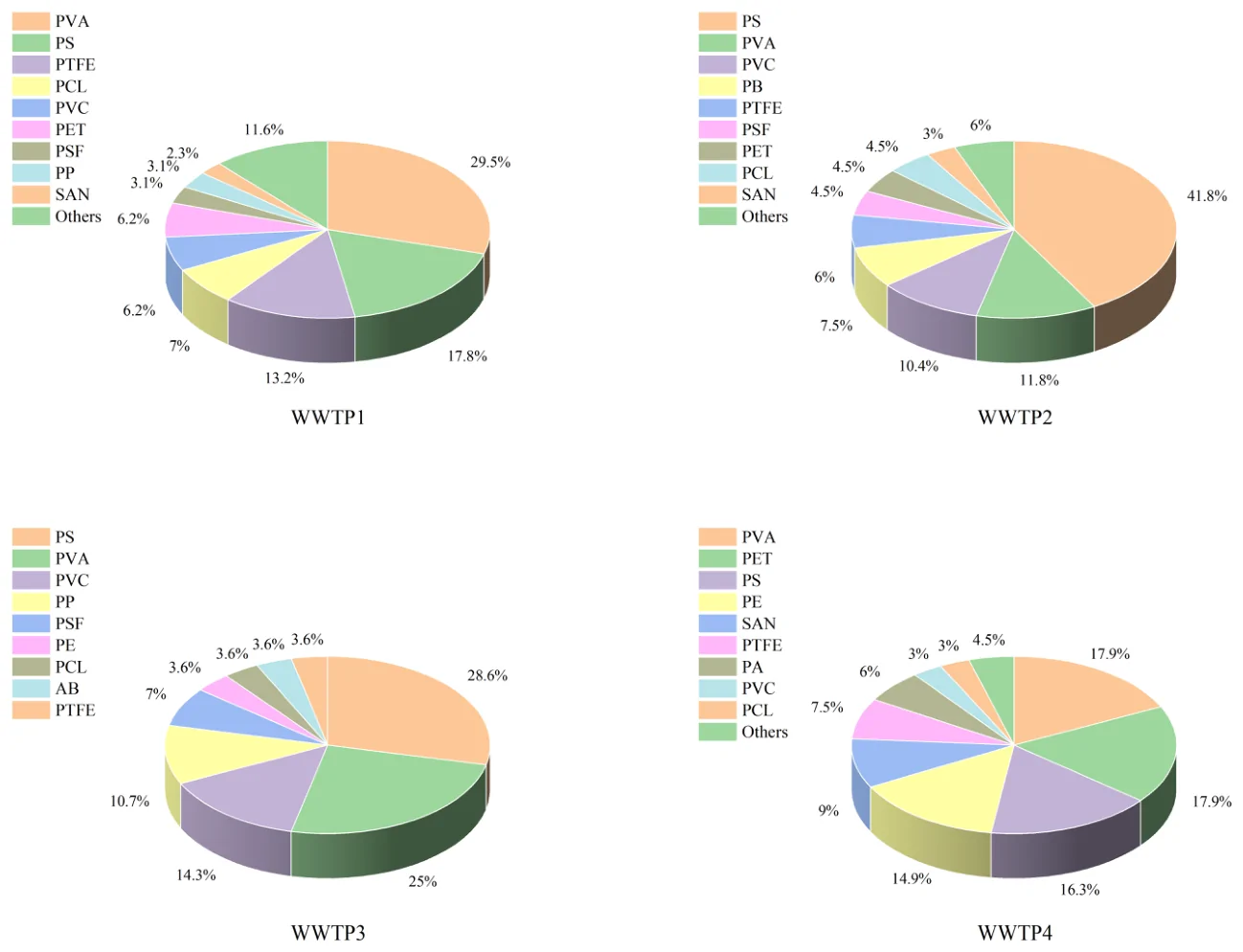
Percentage distribution of each polymer type.

Table 3, Table 4, Table 5 and Table 6 summarize FTIR spectral analysis results of MPs collected from each unit of the four WWTPs, where the total count was obtained using a stereomicroscope. The number of microplastics was confirmed using a Fourier-transform infrared (FTIR) spectrometer. The proportion of MPs particles among total counted particles reached 88.00% (WWTP1), 90.91% (WWTP2), 87.25% (WWTP3) and 80.83% (WWTP4), respectively. The relatively high proportion values were obtained because when the matching degree with the standard samples used in the infrared instrument exceeded 60%, they were therefore classified as such MPs. A total of 13 distinct MPs polymer types were detected in the four WWTPs, and their proportional distributions are illustrated in Figure 2. Additionally, there were still five detected types of MPs with low contents, which were totally indicated as “others”. The units (n·L^−1^) of Total count and MPs meant the number of MPs particles per liter of wastewater.

The polymer compositions of effluent MPs varied markedly across the four WWTPs, exhibiting clear source-related signatures and differential responses to treatment processes. The effluent of WWTP1 was dominated by PVA (29.5%), followed by PS (17.8%) and PTFE (13.2%). WWTP2 had a particularly high proportion of PS (41.8%), with secondary contributions from PVA (11.9%) and PVC (10.4%). For WWTP3, PS (28.6%) and PVA (25.0%) constituted the dominant polymers, accompanied by PVC (14.3%) and PP (10.7%). WWTP4 presented a relatively balanced polymer distribution, with PVA and PET each accounting for 17.9%, plus PS (16.4%) and PE (14.9%). In general, PVA, PS, PET and PVC were the dominant MPs polymers in the effluent of all four facilities.

The divergent polymer distribution among WWTPs was mainly governed by influent pollution source composition and inherent polymer physicochemical properties. PVA possesses strong hydrophilicity and partial water solubility, which tends to form fine colloidal particles that cannot be fully intercepted by conventional sedimentation and filtration units. PS has a low density and high buoyancy; it is prone to fragmentation into fine particles under hydraulic shear, leading to decreased removal efficiency. Polymers including PET, PVC and PTFE exhibit high chemical inertness and cannot be degraded or transformed during biological treatment. Studies published in the past five years have confirmed that although conventional activated sludge processes can achieve overall MPs removal efficiencies above 80%, obvious selective removal differences exist among different polymer types. Polymers with strong hydrophilicity, density close to water or small particle sizes are more likely to escape with final effluent [21,22,23]. In addition, advanced filtration units can effectively intercept large-size PET and PVC particles, yet their removal capacity declines sharply for particles smaller than 100 μm [24,25]. The present results are consistent with previous reports, indicating that MPs polymer composition not only reflects regional pollution input characteristics, but also reveals the inherent limitations of conventional WWTP processes for eliminating MPs with varied physicochemical traits.

### 3.2. Abundance and Removal Efficiency of Microplastics in Each Process Unit of Four Wastewater Treatment Plants

**Figure 3:**
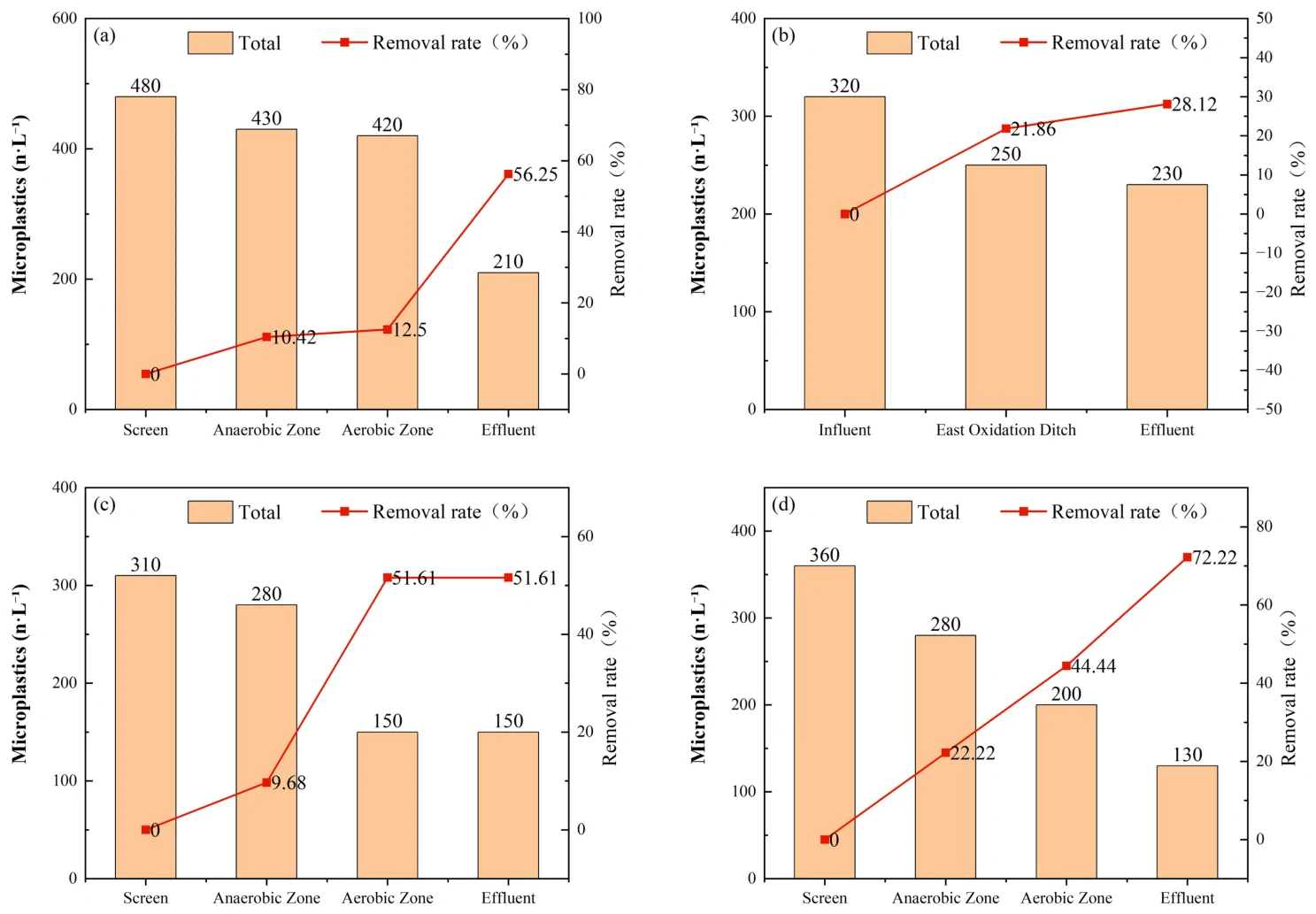
Microplastic abundance and removal efficiency in different wastewater treatment plants (WWTPs). (**a**) Microplastic abundance and removal efficiency in WWTP1; (**b**) Microplastic abundance and removal efficiency in WWTP2; (**c**) Microplastic abundance and removal efficiency in WWTP3; (**d**) Microplastic abundance and removal efficiency in WWTP4.

This study demonstrated that MPs removal during wastewater treatment relies on synergistic effects between biological and advanced treatment units (Figure 3). Biological treatment serves as the fundamental stage for MPs retention, while advanced treatment acts as a critical polishing step to boost overall removal efficiency. Differences in MPs removal performance under different process configurations reflect the combined impacts of hydraulic conditions, sludge floc structure and physical interception mechanisms.

During biological treatment, anaerobic and aerobic tanks initially remove MPs via sludge adsorption, flocculation and sedimentation [3,6]. Taking WWTP1 (Carrousel oxidation ditch) as an example, MPs abundance after bar screening was 480 n·L^−1^. After sequential treatment in anaerobic (430 n·L^−1^) and aerobic zones (420 n·L^−1^), the cumulative removal efficiency rose from 0% to 12.5%, which reflected continuous MPs capture by activated sludge flocs under alternating anaerobic–aerobic environments. WWTP3 (integrated biological tank) displayed outstanding MPs removal capacity, benefiting from high aeration intensity and vigorous microbial activity in the aerobic zone; MPs abundance sharply dropped from 280 n·L^−1^ (anaerobic effluent) to 150 n·L^−1^, corresponding to a removal efficiency of 51.6%. This result highlights the core role of aerobic environments in enhancing MPs entrapment [21]. In contrast, SPND Biopond contributed moderately to MPs elimination in WWTP2, with total biological removal efficiency increasing from 0% to 21.9%. This may be associated with the high proportion of low-density, buoyant polystyrene (PS) MPs in its influent, and relevant literature also confirms that low-density polymers easily penetrate conventional activated sludge systems [22].

The advanced treatment stage includes units between the aerobic tank outlet and final disinfection, including secondary sedimentation tanks, high-efficiency coagulation tanks, fiber rotary disc filters, denitrifying deep bed filters and disinfection units, which significantly strengthen MPs sieving and interception [24,25]. In WWTP4 (CASS process), MPs abundance of 200 n·L^−1^ after aerobic treatment decreased to 130 n·L^−1^ after sequential treatment by secondary sedimentation, mixed reaction sedimentation and denitrifying deep bed filtration. The overall removal efficiency increased from 44.4% to 72.2%, among which deep bed filtration played the dominant role in intercepting residual fine MPs. Advanced treatment also generated remarkable improvement in WWTP1, lifting the total removal rate from 12.5% to 56.3%, and fiber rotary disc filters enhanced MP elimination via physical screening and surface adsorption [12]. However, advanced treatment failed to further reduce MPs abundance in WWTP3, with effluent MPs concentration remaining unchanged. This outcome may be attributed to secondary MPs release and resuspension induced by elevated hydraulic shear during magnetic coagulation and rotary drum filter operation [23]. Advanced treatment exerted limited effects in WWTP2, only raising the total removal efficiency from 21.9% to 28.12%, which further verifies that influent polymer characteristics regulate subsequent treatment performance.

In summary, biological treatment units realize primary MPs removal through sludge floc adsorption, entrapment and gravitational settling, while advanced treatment facilities substantially improve removal efficiency via physical interception and enhanced solid-liquid separation. Variable removal performance across different processes suggests that biological operational parameters should be optimized, and advanced treatment configurations should be strengthened according to the dominant MPs types and physicochemical properties in the influent, so as to elevate overall MPs removal efficiency and effluent quality.

### 3.3. Removal Performance of Different Process Flows for Microplastics with Various Shapes

**Figure 4:**
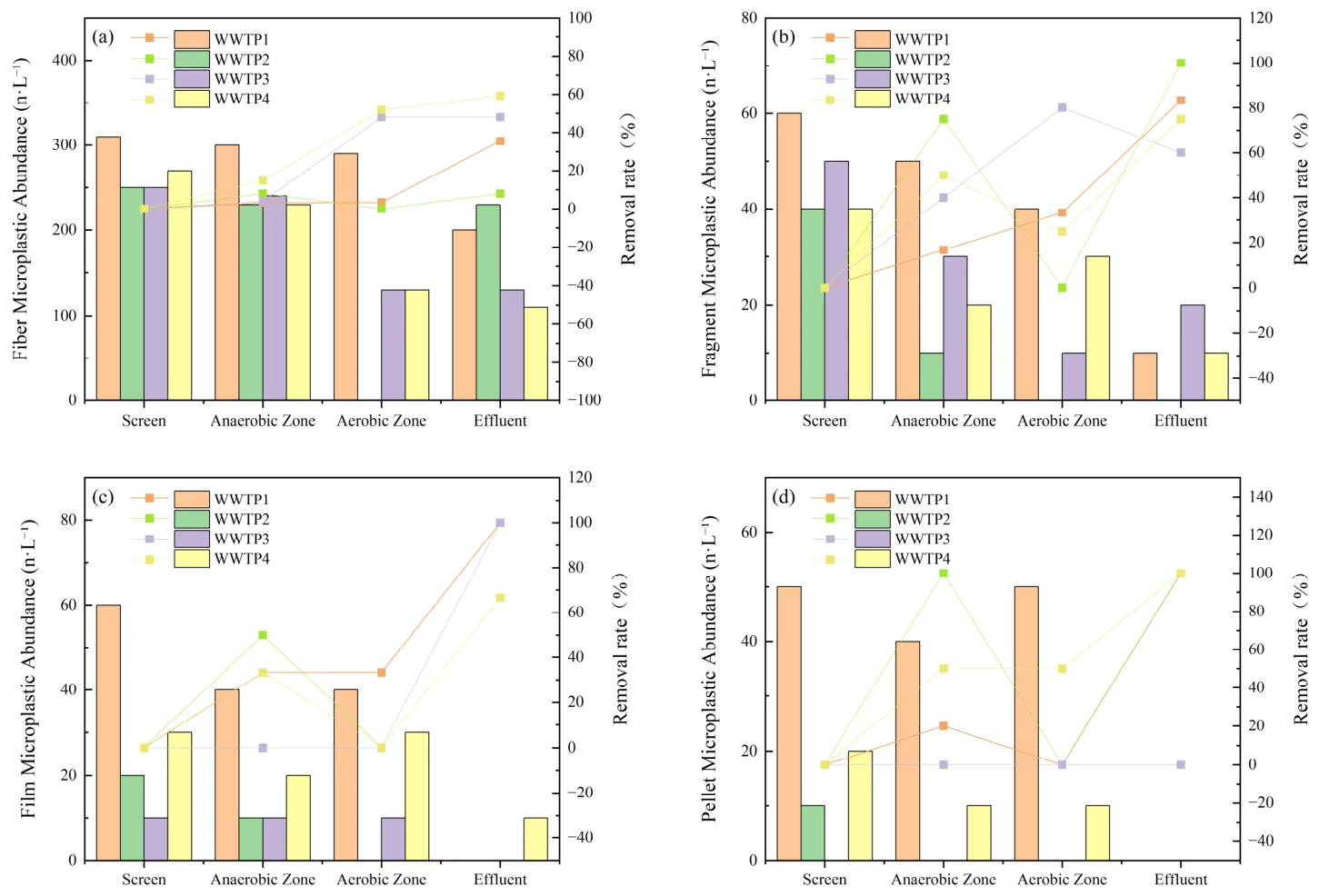
(**a**) Fiber microplastics; (**b**) Fragment microplastics; (**c**) Film microplastics; (**d**) Pellet microplastics.

The removal of microplastics (MPs) with different morphologies was jointly controlled by their inherent physicochemical properties and wastewater treatment processes, leading to clear shape-dependent removal patterns (Figure 4) [26,27]. Fibrous MPs were mainly retained via sludge adsorption and physical interception in advanced filtration facilities [28]. WWTP4, equipped with a CASS process coupled with a deep bed filter, attained a fiber removal efficiency of 59.3%, whereas insufficient interception capacity resulted in only 8% fiber removal in WWTP2. Hydraulic disturbance and filter backwashing triggered the resuspension of fibrous MPs in WWTP3, which aligned with prior research reporting aeration-induced resuspension of fibrous microplastics [29]. High-density fragmented MPs preferentially settled through gravitational sedimentation [30]. WWTP1 (Carrousel oxidation ditch + rotary disc filter) achieved a fragment removal efficiency of 83.3%. Although aeration-induced particle resuspension occurred in WWTP2 and WWTP3, their advanced treatment facilities intercepted most fragmented MPs, and the deep bed filter of WWTP4 realized 75% removal of residual fragments. Low-buoyancy film-shaped MPs cannot settle independently in conventional biological treatment systems [26]. WWTP1–WWTP3 achieved complete removal of film-shaped MPs through coagulation or deep filtration; however, influent concentration fluctuations and filter clogging led to no observable film MPs removal in WWTP4, as ultra-thin film particles readily penetrate filter layers under high flow velocity [12].

In summary, fragments and pellets were primarily eliminated in secondary sedimentation tanks, while fibers and films relied on enhanced coagulation and advanced filtration for effective removal. Optimizing aeration intensity, sludge reflux ratio and filter backwashing parameters could realize targeted control of MPs, which was consistent with the conclusions of existing studies.

### 3.4. Removal Performance of Different Process Flows for Microplastics with Various Colors

**Figure 5:**
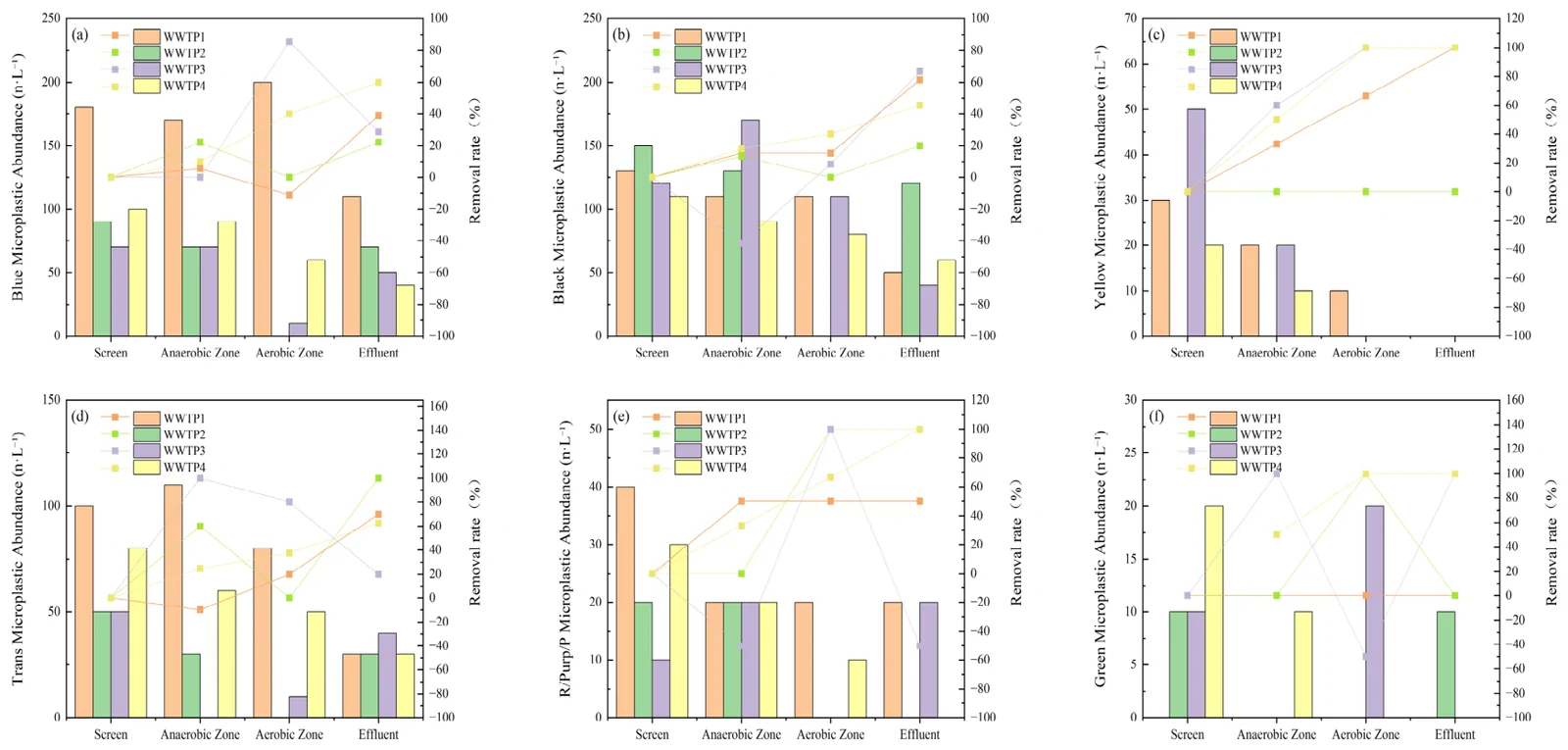
Removal rate of microplastics with different colors. (**a**) Blue microplastics; (**b**) Black microplastics; (**c**) Yellow microplastics; (**d**) Transparent microplastics; (**e**) Red/Purple microplastics; (**f**) Green microplastics.

MPs removal efficiency varied significantly with MPs color, which was simultaneously governed by MPs source characteristics, intrinsic physicochemical properties and the removal mechanisms of different wastewater treatment units [26,27]. It can be seen in Figure 5. For dark-colored MPs, blue MPs achieved a maximum removal efficiency of approximately 90% in WWTP3 through sludge adsorption and subsequent advanced filtration, and the overall MPs removal efficiency of this plant reached 51.61%. In comparison, WWTP1 and WWTP2 showed low removal rates for blue MPs, and hydraulic disturbance and sludge recirculation triggered blue MPs resuspension in WWTP4 [29]. Black MPs maintained stable removal across all four WWTPs, with removal efficiencies exceeding 50% in WWTP1 and WWTP2 due to their high density and favorable settling performance with sludge flocs. Biological treatment induced minor fluctuations in black MP removal in WWTP3 and WWTP4, yet advanced treatment units effectively trapped residual black MPs in both plants.

Light-colored MPs showed clear process-dependent differences in removal. Low-density, buoyant yellow MPs originating from packaging materials achieved nearly complete removal in WWTP3 and WWTP4, but only attained 30% removal efficiency in WWTP1 and WWTP2, which highlighted the indispensable role of advanced filtration for such MPs [12]. Transparent MPs reached an 80% removal rate in WWTP1, yet aeration shear force induced their fragmentation and accumulation in the aerobic zones of WWTP2 and WWTP3, and only WWTP4 maintained stable removal performance for transparent MPs [31]. Red/purple MPs containing chemically stable industrial dyes were prone to resuspension under hydraulic disturbance. These MPs presented steady removal in WWTP1 but generated net MPs release in WWTP3 and WWTP4 [32].

In summary, dense dark-colored MPs with strong sludge affinity were primarily removed via biological sedimentation, whereas low-density and light-colored MPs were susceptible to fragmentation and required physical sieving as well as filtration in advanced treatment units for effective elimination. Although the association between color and density (dark MPs = higher density) is a generalization that does not always hold true, the commonly used plastic materials and colors are generally quite stable. So, such color-based removal discrepancies indicated that targeted optimization of aeration intensity, sludge reflux ratio and filtration parameters were based on MPs color; meanwhile, pollution sources and physical traits can effectively improve the overall MPs removal efficiency of WWTPs.

### 3.5. Removal Performance of Different Process Flows for Microplastics with Various Sizes

**Figure 6:**
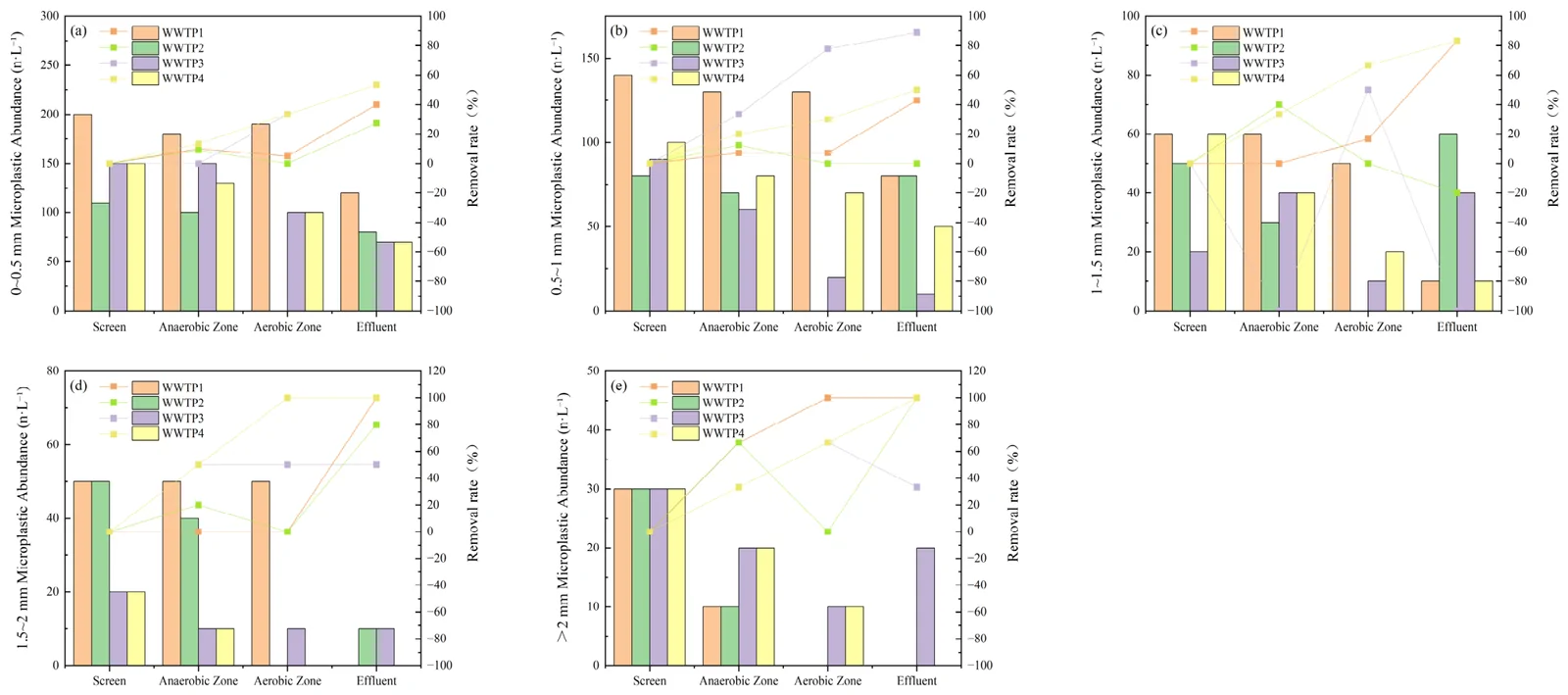
Removal rate of microplastics with different sizes. (**a**) 0–0.5 mm, (**b**) 0.5–1 mm, (**c**) 1–1.5 mm, (**d**) 1.5–2 mm, (**e**) >2 mm.

The removal of MPs with different particle sizes exhibited obvious size-dependent characteristics during wastewater treatment, which was mainly governed by activated-sludge flocculation, sedimentation, and advanced filtration of activated sludge flocculation and sedimentation, as well as the physical interception capacity of advanced treatment units [26,27]. As it is shown in Figure 6, Fine MPs (<0.5 mm) were dominant in all water samples and were the most difficult fraction to retain. WWTP3 achieved approximately 90% removal efficiency for fine MPs through aerobic flocculation combined with deep filtration. By contrast, WWTP1 and WWTP2 had weaker interception capacity for fine MPs, and sludge recirculation triggered the resuspension and an increase in small-particle concentrations in the aerobic tank of WWTP4 [29]. Medium-sized MPs (0.5–1 mm) followed a similar removal trend: WWTP1–WWTP3 achieved favorable removal performance via sedimentation and filtration, while WWTP4 had low removal efficiency. Large MPs (1–1.5 mm) gradually declined in abundance throughout the treatment sequence and were almost completely eliminated in WWTP3 and WWTP4. Aeration-induced fragmentation caused concentration fluctuations of large MPs in the aerobic zones of WWTP1 and WWTP2. Extra-large MPs (>1.5 mm) readily settle in secondary sedimentation tanks [3]. WWTP1 realized excellent elimination of extra-large MPs, while unstable MPs concentrations in the biological stages of WWTP2 and WWTP3 were offset by subsequent advanced treatment.

In summary, particle size constitutes one of the core factors controlling MPs removal behavior. Fine MPs (<0.5 mm) should be prioritized for control owing to their high proportion and strong migration capacity, and their removal relied heavily on advanced filtration and enhanced flocculation measures. Although large MPs (>1 mm) settled easily, they faced fragmentation risks under aeration shear; therefore, the optimization of aeration intensity and filter operating parameters was still required to avoid secondary MPs release. The process configuration of each WWTP exerted a decisive influence on the elimination of MPs of different size fractions. Specifically, WWTP3 delivered superior removal performance for MPs of all size ranges by integrating high-efficiency biological adsorption and advanced filtration, whereas WWTP4 required further operational optimization to address the resuspension of large-sized MPs. The above results provided a theoretical basis for optimizing wastewater treatment processes according to size-based MP characteristics to achieve precise MP control.

### 3.6. Effects of Process Operational Parameters on MPs Removal Efficiency

**Figure 7:**
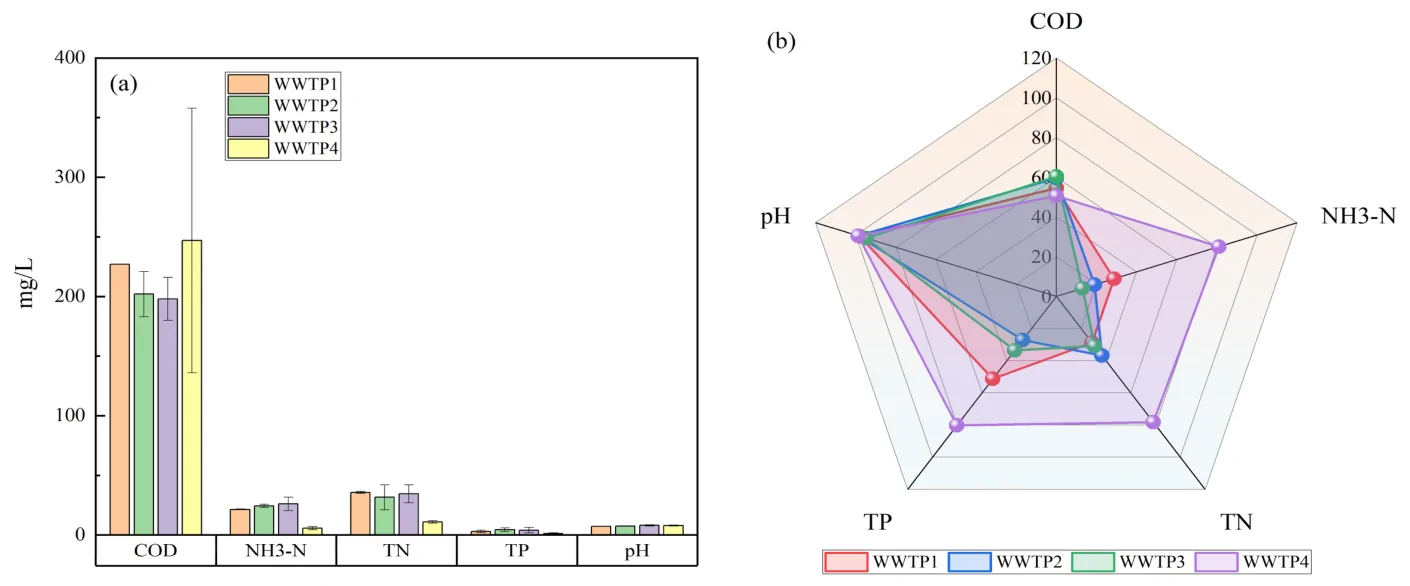
Comparison of influent water quality indices in four wastewater treatment plants (WWTPs). (**a**) Bar charts of the mean values and standard errors of COD, NH_3_-N, TN, TP and pH in each WWTP; (**b**) Normalized radar chart of water quality indices, where values closer to 100 indicate better water quality (lower concentrations of pollution indices and pH closer to neutral).

Figure 7a,b systematically illustrated the influent water quality profiles and normalized comprehensive operational performance of the four WWTPs, providing critical operational background for analyzing the disparities in MP removal efficiency. Recent studies confirmed that influent pollution load and its fluctuation indirectly regulate MPs migration and fate in biological treatment systems by altering sludge floc structure, microbial activity and settling performance [10,33,34]. Here, all the water quality indicator data were provided by WWTPs.

In terms of raw water quality indicators (Figure 7a), significant differences existed in influent pollutant loads across the four facilities. High organic loads strengthened the encapsulation of MPs by sludge flocs, yet large load fluctuations may destroy the stability of sludge floc structure [33]. The ranking of NH_3_-N concentration was WWTP3 > WWTP2 > WWTP1 > WWTP4, and WWTP3 had the highest ammonia nitrogen load (26.1 mg/L). A high ammonia nitrogen environment reduced floc compactness, thereby weakening the adsorption and sedimentation efficiency of MPs [33]. WWTP1 and WWTP3 exhibited relatively high TN concentrations, while WWTP4 had a far lower TN level (10.9 mg/L). Low nitrogen loads helped maintain stable nitrification–denitrification processes and preserve the structural integrity of sludge flocs. WWTP2 contained the highest TP concentration (4.37 mg/L). Excessive phosphorus may trigger sludge bulking, impair sludge settling performance and raise the risk of MP discharge in final effluent [34]. The pH values of all influent samples ranged from 7.18 to 8.0, consistent with the weakly alkaline characteristics of municipal domestic sewage. Among the four plants, WWTP1 maintained the most stable pH, whereas WWTP3 experienced prominent pH fluctuations (standard error = 0.6). The pH variation altered microbial metabolism and extracellular polymeric substance (EPS) secretion, further affecting MPs flocculation and encapsulation efficiency [35].

The normalized radar chart (Figure 7b) further revealed the comprehensive stability of influent water quality of each WWTP. WWTP4 presented high normalized values for NH_3_-N, TN and TP with mild overall fluctuations and balanced water quality composition, forming a relatively stable operating environment for activated sludge systems. Such stable conditions promoted the formation of compact sludge flocs and improved biosorption and sedimentation of MPs, corresponding to its maximum total MPs removal efficiency of 63.89%. It could also be inferred that the stable wastewater conditions may be due to the hydrolysis-acidification pretreatment process in WWTP4, and this process only existed in WWTP4. Although WWTP1 had a relatively high TN load, its stable pH maintained consistent SRT and intact microbial community structure, thus guaranteeing moderate MPs removal capacity. WWTP2 displayed large fluctuations in TP and TN, indicating unstable influent conditions that disrupt sludge settling performance, which matched its relatively low MPs removal efficiency (28.12%). WWTP3 carried the highest ammonia nitrogen load and obvious fluctuations in multiple water quality indicators. Frequent pollutant load shocks damage sludge floc structure and trigger the resuspension or secondary release of adsorbed MPs [36].

Overall, stable influent water quality acted as an essential prerequisite for high MPs removal efficiency. Benefiting from balanced pollutant concentrations and mild fluctuations, WWTP4 created favorable conditions for biological and advanced treatment units and achieved optimal MPs removal performance. High ammonia or phosphorus loads and severe water quality fluctuations in WWTP2 and WWTP3 destroyed sludge structural stability and microbial activity, weakening the adsorption and sedimentation capacity for MPs. WWTP1 maintained moderate MPs removal efficiency under steady pH conditions.

### 3.7. Mechanism Analysis of Differences in MPs Removal Performance Among Different Treatment Processes

By integrating the migration and removal behaviors of MPs with diverse polymer types, colors and particle sizes in the four WWTPs, it could be concluded that MPs removal was synergistically co-determined by MPs physicochemical properties and the operational conditions of wastewater treatment systems. Polymers with high chemical stability, strong hydrophobicity or low density (e.g., PS, PTFE, PP) exhibited strong penetrability in conventional biological treatment units, and their removal efficiencies were generally lower than those of high-density polymers with strong sludge-binding affinity. This phenomenon was mainly because low-density polymers readily floated and were susceptible to hydraulic disturbance, which reduced their sedimentation and adsorption probability [37,38].

As a comprehensive reflection of MPs physical properties, color also revealed disparities in pollution sources and particle density. Dark-colored MPs (e.g., blue, black) generally achieved higher overall removal efficiencies than light-colored MPs (e.g., yellow, transparent) due to their higher density and stronger affinity with sludge flocs. This observation agreed with recent research showing that high-density MPs were easily removed via sludge floc adsorption and gravitational settling, while low-density or highly hydrophobic MPs tended to escape into final effluent [29,39]. In addition, light-colored MPs were prone to fragmentation into fine particles under aeration shear force, further increasing the difficulty of interception and removal.

Particle size was another critical factor governing MPs removal performance, following a universal trend that removal efficiency increased with particle size. Fine MPs (especially 0–0.5 mm) were easily suspended and tended to migrate or fragment under aeration shear, making them the most challenging fraction to control. Small-sized MPs responded weakly to biological flocculation and primary sedimentation, and their elimination heavily relied on the physical interception capacity of advanced treatment units [34,40]. In contrast, relatively large MPs (>1 mm) could achieve high removal rates in biological treatment stages due to their high density and favorable settling performance. However, large MPs faced fragmentation risks under high-intensity aeration, and such particle fragmentation had been widely observed in various treatment processes [3].

Process operational parameters exerted fundamental regulatory effects on MPs removal. As an enhanced interception barrier, advanced treatment units played a decisive polishing role for fine particle MPs, and the selection of treatment facilities significantly affected final MPs removal efficiency. Compared with processes relying solely on sedimentation, filtration-based advanced treatment (e.g., fiber rotary disc filters, deep bed filters) achieved superior physical interception of fine particle MPs [24,41,42], which was supported by the effluent performance data of all four WWTPs in this study.

## 4. Conclusions

Differences in overall MPs removal performance were observed among the four WWTPs equipped with different process flows. WWTP4 achieved the highest comprehensive removal efficiency, followed by WWTP1 and WWTP3, while WWTP2 exhibited the weakest elimination capacity. Based on unit-by-unit MPs abundance variations and total removal rates, WWTP4 attained the highest overall MPs removal efficiency of 63.89% via the combined action of biological treatment and denitrifying deep bed filtration, showing prominent advantages in removing fragmented, large-size and some fibrous MPs. Benefiting from stable operation and enhanced interception by fiber rotary disc filters, WWTP1 presented favorable comprehensive MPs removal performance. WWTP3 realized outstanding removal of fine MPs in aerobic tanks, yet its overall performance was inferior to WWTP4 due to unstable influent water quality and limited advanced treatment capacity. Restricted by high phosphorus load and drastic water quality fluctuations, WWTP2 showed the lowest total MPs removal efficiency (28.12%). These results demonstrated that the combined system of stable biological treatment plus high-efficiency advanced filtration was a promising strategy for MPs control under current municipal wastewater treatment conditions, which were consistent with previous studies confirming that deep bed filters and fine filtration possess superior advantages for enhanced MPs removal.

## Figures and Tables

**Table 1:** Experiment reagents.

Reagent Name	Molecular Formula/Abbreviation	Purity/Specification	Manufacturer (City, Country)
30% Hydrogen peroxide	H_2_O_2_	30%	Tianjin Kermel Chemical Reagent Co., Ltd., Tianjin, China
Anhydrous ethanol	C_2_H_5_OH	Analytical grade	Tianjin Fuyu Fine Chemical Co., Ltd., Tianjin, China
Sodium chloride	NaCl	Analytical grade	Yantai Shuangshuang Chemical Co., Ltd., Yantai, China
Liquid nitrogen	N_2_	High-purity liquid nitrogen	Henan Keyi Gas Co., Ltd., Henan, China
Glass fiber filter membrane	-	1 μm	Shanghai Xingya Purification Material Factory, Shanghai, China

**Table 2:** Experiment instruments.

Instrument Name	Model	Manufacturer (City, Country)
Analytical balance	BNT224N	Bonita Scientific Instrument Co., Ltd., Nanjing, China
Electric heating blast constant-temperature drying oven	DHG-9246A	Shanghai Jinghong Experimental Equipment Co., Ltd., Shanghai, China
Circulating water multi-purpose vacuum pump	SHB-IIIA	Zhengzhou Yarong Instrument Co., Ltd., Zhengzhou, China
Centrifuge	CenLee4K	Hunan Xiangli Scientific Instrument Co., Ltd., Hunan, China
Constant-temperature incubator shaker	SHA-C 5	Changzhou Zhiborui Instrument Manufacturing Co., Ltd., Changzhou, China
Portable multi-parameter tester	ULTRAMETER 6PFC	Myron L Company, Carlsbad, CA, USA
Fourier-transform infrared microspectrometer	Thermo Fisher Nicolet iN10 MX	Thermo Fisher Scientific Inc., Waltham, MA, USA
Stereomicroscope	OST-SZN6745	Suzhou Ouster Optical Instrument Co., Ltd., Suzhou, China
Ultrasonic cleaner	KS-5200DE	Kunshan Jielimei Ultrasonic Instrument Co., Ltd., Kunshan, China

**Table 3:** WWTP1 Spectroscopic analysis results statistics.

WWTP1	Total Count (n·L^−1^)	Microplastics (n·L^−1^)	Proportion (%)
Coarse Screen	560	480	85.71
Anaerobic	440	430	97.73
Aerobic	510	420	82.35
Effluent	240	210	87.50
Total	1750	1540	88.00

**Table 4:** WWTP2 Spectroscopic analysis results statistics.

WWTP2	Total Count (n·L^−1^)	Microplastics (n·L^−1^)	Proportion (%)
Coarse Screen	330	320	96.97
SPND Biopond	300	250	83.33
Effluent	250	230	92.00
Total	880	800	90.91

**Table 5:** WWTP3 Spectroscopic analysis results statistics.

WWTP3	Total Count (n·L^−1^)	Microplastics (n·L^−1^)	Proportion (%)
Coarse Screen	360	310	86.11
Anaerobic	320	280	87.50
Aerobic	180	150	83.33
Effluent	160	150	93.75
Total	1020	890	87.25

**Table 6:** WWTP4 Spectroscopic analysis results statistics.

WWTP4	Total Count (n·L^−1^)	Microplastics (n·L^−1^)	Proportion (%)
Coarse Screen	360	360	100.00
Anaerobic	400	280	70.00
Aerobic	240	200	83.33
Effluent	200	130	65.00
Total	1200	970	80.83

## Data Availability

The original contributions presented in this study are included in the article Materials. Further inquiries can be directed at the corresponding author.

## Abbreviations

The following abbreviations are used in this manuscript:

AB	Acrylonitrile Butadiene Styrene
A^2^/O	Anaerobic-Anoxic-Oxic
CASS	Cyclic Activated Sludge System
COD	Chemical oxygen demand
HRT	Hydraulic retention time
MPs	Microplastics
PA	Polyamide
PB	Polybutylene
PCL	Polycaprolactone
PE	Polyethylene
PET	Polyethylene terephthalate
PP	Polypropylene
PS	Polystyrene
PSF	Polysulfone
PTFE	Polytetrafluoroethylene
PVA	Polyvinyl Alcohol
PVC	Polyvinyl chloride
SAN	Styrene Acrylonitrile resin
SBR	Sequencing Batch Reactor
TN	Total nitrogen
TP	Total phosphorus
WWTPs	Wastewater treatment plants
